# Depression scores and its relationship with sexual quality of life in women with type 1 and type 2 diabetes: A cross-sectional study

**DOI:** 10.1097/MD.0000000000038641

**Published:** 2024-08-09

**Authors:** Ekin Dila Topaloğlu Ören, Elif Ünsal Avdal, Funda Sofulu, Gökşen Polat, Gönül Düzgün, Gülseren Pamuk

**Affiliations:** a Department of Obstetrics and Gynecology Nursing, Izmir Katip Celebi University Faculty of Health Science, İzmir, Türkiye; b Department of Internal Medicine Nursing, Izmir Katip Celebi University Faculty of Health Science, İzmir, Türkiye; c Department of First Aid and Emergency, Izmir Tinaztepe University Faculty of Health Science, İzmir, Türkiye; d Department of Internal Medicine Nursing, İzmir Tinaztepe University Faculty of Health Science, İzmir, Türkiye; e Department of Family Medicine, Izmir Katip Celebi University School of Medicine, İzmir, Türkiye.

**Keywords:** depression, diabetes, quality of sexual life, sexual life, women

## Abstract

Diabetes is an important public health problem with increasing prevalence worldwide. However, the prevalence of diabetes in women is increasing. Women with diabetes have many physical and psychological complications. It has been reported that complications experienced by women with diabetes negatively affect both their sexual and mental health. This study aimed to determine the sexual quality of life (SQoL) and depression scores in women with type 1 diabetes (T1D) and type 2 diabetes (T2D), the relationship between them, and to examine the factors predicting the SQoL. This study was analytical and cross-sectional. This study was conducted with 440 women with diabetes (206 women with type 1 and 234 women with type 2 diabetes) who came to the endocrine policlinic of a university hospital in Izmir, western Türkiye, between April and October 2023. Data were collected using the “Individual Description Form,” “Sexual Quality of Life Questionnaire” and “Beck Depression Inventory.” Correlation and multiple regression analyses were conducted to investigate the relationship between SQoL and depression scores. When women with T1D and T2D were compared, it was determined that women with T2D had worse SQoL and higher depression scores (*P* < .05). Both T1D and T2D women were found to have a strong negative correlation between SQoL and depression scores (*r* = −0.753; −0.837; *P* < .05). Age (*B* = −0.291), body mass index (BMI; *B* = −2.747), type 2 diabetes (*B* = −3.074), and depression scores (*B* = −1.898) were predictive factors of SQoL in women with diabetes (*R*^2^ = 0.670; *P* < .05). In our study, it was determined that depression scores were increased in women with diabetes mellitus with decreased SQoL. When T1D and T2D were compared, T2D had worse SQoL and higher depression scores. It also revealed that age, BMI, T2D, and depression scores affected SQoL. Healthcare professionals especially nurses should provide education and counseling to women with T1D and T2D about sexual life and mental health.

## 1. Introduction

Diabetes is a chronic metabolic disorder characterized by persistent hyperglycemia. Prolonged elevation of blood glucose levels associated with this condition can result in significant pathological damage to various organ systems, including the cardiovascular system, vasculature, retina, renal structures, and peripheral nerves. Diabetes is classified as type 1 diabetes (T1D), type 2 diabetes (T2D), and gestational diabetes. T1D is a chronic autoimmune disorder characterized by the destruction of insulin-producing beta cells in the pancreas, resulting in minimal or absent endogenous insulin production. T2D is a disease that occurs when the body becomes resistant to insulin or cannot produce enough insulin.^[1]^ Over the last 30 years, the prevalence of T2D has been increasing dramatically in countries of all income levels. Approximately 422 million people worldwide have diabetes, the majority of whom live in low- and middle-income countries, and 1.5 million deaths each year are directly attributable to diabetes. Both the number of cases and the prevalence of diabetes have been increasing steadily over the last few decades. According to the International Diabetes Federation (IDF), the global prevalence of diabetes was 415 million individuals in 2015. This figure is projected to increase to 612 million by 2040.^[1,2]^ According to the results of the Turkish Diabetes Epidemiology (TURDEP I-II) study, the prevalence of diabetes increased from 7.2% to 13.7%.^[3,4]^ Obesity, advanced age, family history, low socio-economic status, sedentary life, genetic predisposition, smoking, excessive calorie consumption, and increase in body mass index (BMI) were associated with increased susceptibility to diabetes.^[2,5,6]^ When diabetes risk factors were taken into consideration, it was reported that the prevalence of diabetes was increasing every year in women compared to men (especially T2D).^[6–8]^ Women were more exposed to many physical (ketoacidosis, hypoglycemia, frequent urination, damage to nerves as well as vessels, impotence, etc)^[9,10]^ and psychological health problems (anxiety, depression, low self-esteem, etc) caused by both the physiological cycle of being a woman and diabetes.^[11–13]^ Among the physical and psychological health problems experienced by diabetic women, sexual and psychological health problems were at the top of the list.^[11,14,15]^ Previous studies on the sexual health of women with diabetes focused on sexual dysfunctions experienced by them^[14–18]^ and there were very few studies in the literature in which sexual quality of life (SQoL) was measured.^[19–23]^ However, SQoL was an important parameter including sexual health and sexual functions.^[24,25]^ For this reason, we have evaluated the SQoL in women with diabetes in our study. On the other hand, the prevalence of depression in individuals with diabetes varied within a wide range of 10.9% to 67.5%, and the prevalence of depression continued to increase in women with diabetes due to diabetes and changes in living conditions (socio-demographic factors, unhealthy diet, sedentary life, problems with body image and self-esteem).^[26–33]^ Despite the increasing prevalence of sexual health problems and depression among women with diabetes, considerations of SQoL and depression levels are often deprioritized during the diagnostic, treatment, and care processes, with the primary focus being on managing the disease and its side effects.^[5,6,8]^ As a result, the SQoL and depression levels of women with diabetes are frequently relegated to secondary importance or even ignored. Although the type of diabetes significantly impacts the SQoL and depression levels in women, there is a notable scarcity of studies that evaluate these factors in relation to T1D and T2D.^[19–21,34]^ This study aims to underscore the importance of these issues to all healthcare professionals, particularly diabetes nurses. It seeks to highlight the need for integrating assessments of SQoL and depression levels into all stages of diabetes management, including diagnosis, treatment, and ongoing care, thereby ensuring a more comprehensive approach to the well-being of women with diabetes.^[35,36]^

This study aims to improve the early diagnosis and management of sexual and psychological health issues in women with diabetes by evaluating their SQoL and depression levels according to diabetes type. Enhancing these aspects of health will improve treatment efficacy and minimize side effects. Unlike previous research, our study comprehensively examines these variables in women with T1D and T2D, revealing their interrelationships and influencing factors. The objectives are to determine SQoL and depression scores in women with T1D and T2D, explore their relationship, and identify predictors of SQoL. To improve the sexual and psychological health of women with diabetes, we recommend: providing evidence-based information; using early assessment tools for sexual and psychological health; identifying and addressing related issues; and developing coping strategies. Additionally, regular education and counseling by diabetes nurses are advised. These measures will significantly enhance the sexual and psychological health of women with diabetes.

## 2. Materials and methods

### 2.1. Design

This study was analytical and cross-sectional. The study adhered to the Strengthening the Reporting of Observational Studies in Epidemiology (STROBE) Statement, which provides guidelines for reporting observational studies.^[37,38]^

### 2.2. Study setting and sampling

This analytical and cross-sectional study was conducted in the endocrine outpatient clinic of a university hospital (*where the hospital was a public institution*) in Izmir, in western Türkiye, to identify SQoL and depression scores for women with T1D and T2D who came for routine control, between April and October 2023.

*The study population* comprised 1276 women with diabetes who visited an endocrine outpatient clinic for routine checkups between January and December 2022. Aiming for a 95% confidence interval and a 5% type I error rate, a sample size of 296 women was calculated using the formula for selecting a known population. However, *the final sample* included 440 women who met the inclusion criteria and volunteered to participate between April and October 2023 at a university hospital in Izmir, located in western Türkiye. No sample limitation was imposed, and the entire population meeting the criteria within the specified dates was included. Data collection occurred every weekday, employing purposive sampling, a non-probability sampling method.

In the study, *267 women with T1D were invited*. Of these women, 20 were excluded because they had chronic diseases other than diabetes, 11 were illiterate, 3 had sexual dysfunction, 3 could not speak Turkish, 12 were not sexually active, 5 did not complete the surveys and 7 did not accept to participate in the study. In our study, *298 women with T2D were invited.* Of these women, 16 were excluded because they had chronic diseases other than diabetes, 5 were illiterate, 5 had sexual dysfunction, 6 could not speak Turkish, 15 were not sexually active, 7 did not complete the questionnaires and 10 did not accept to participate in the study. *The final study sample comprised 440 women with diabetes, including 206 with type 1 diabetes and 234 with type 2 diabetes. Among those invited to participate, 77.2% of the women with type 1 diabetes and 78.5% of the women with type 2 diabetes agreed to be included in the study.*

Based on the outcome of the post hoc power analysis, the study’s power, calculated with a 5% margin of error and a correlation coefficient of −0.753 for the association between quality of sexual life and depression in women with diabetes, was determined to be 99.9%. This indicates that the study’s power is deemed acceptable, suggesting that the dataset is adequately sized.^[39]^

### 2.3. Inclusion and exclusion criteria

The inclusion criteria for the study were as follows: women who visited the endocrine outpatient clinic for routine checkups, did not have any chronic disease other than diabetes, had no history of psychiatric illness, were at least literate, could speak Turkish, were sexually active, and agreed to participate in the study. The exclusion criteria included women with newly diagnosed diabetes, those referred to the endocrine outpatient clinic from other outpatient clinics, women who had not been sexually active for the past 6 months, those diagnosed with sexual dysfunction or psychiatric illness, those who incompletely completed the surveys, and those who did not consent to participate in the study.

### 2.4. Instrument

Data were collected using the “Individual Description Form,” “Beck Depression Inventory (BDI),” and “Sexual Quality of Life Questionnaire (SQOL).” Participants completed these forms through face-to-face interviews, which took approximately 20 minutes each.

#### 2.4.1. Individual description form

The Individual Description Form was developed by the researchers drawing upon prior studies.^[4–6]^ The form comprised 9 questions addressing sociodemographic characteristics, type of diabetes, body mass index, and the number of living children among women with diabetes.

#### 2.4.2. Beck depression inventory (BDI)

The original version of the scale was developed by Beck et al^[40]^ and later adapted for Turkish use by Hisli,^[41]^ who conducted its validity and reliability study. Participants were asked to select a statement reflecting their feelings over the past week, including the day of data collection. Responses were scored on a scale of 0 to 3, with a possible total score range of 0 to 63. A cutoff score of 17 or higher indicated a need for depression treatment in 90% of patients. Higher scores indicated increased depression levels, approaching a maximum score of 63. The scale demonstrated good internal consistency, with a Cronbach alpha value of 0.80 in previous studies^[40,41]^ and 0.78 in the current study.

#### 2.4.3. Sexual quality of life questionnaire (SQOL)

The scale was developed by Symonds et al^[42]^ to assess women’s SQoL, with its Turkish validity and reliability established by Tuğut and Gülbaşi.^[43]^ The scale, a 6-point Likert type, consists of 18 items, with scores ranging from 18 to 108. Scores are converted to a scale of 0 to 100 using the formula: (crude score −18) × 100/90. Higher scores indicate better SQoL, with a maximum score of 100. The SQOL was utilized in this study to evaluate the SQoL of women with diabetes, as it has been previously used in both healthy women and those with diabetes in Türkiye. The Turkish version of the SQOL includes items such as perceptions of sexual life enjoyment, anger, and concerns about the future. This scale has been applied not only in cohorts of healthy women but also in populations of women with diabetes in Türkiye.^[19,23]^ The scale demonstrates good internal consistency, with a Cronbach alpha coefficient of 0.75 in previous studies^[43]^ and 0.81 in the current study.

### 2.5. Data collection

After obtaining ethical approval from both the university and the study hospital, the primary researcher contacted the relevant nursing departments to secure their support. Subsequently, the researcher met with the head nurses of the hospital’s endocrine outpatient clinic and obtained their consent for the study. Women diagnosed with diabetes who visited the clinic between April and October 2023 were interviewed. The researchers provided a comprehensive overview of the study’s objectives, potential advantages, and anticipated time requirements to the participants. Subsequently, verbal and written informed consent were obtained from each participant, ensuring their full understanding and voluntary participation in the study. After consenting, participants completed an individual identification form, the Turkish version of the BDI, and the SQOL, which took approximately 20 minutes. Researchers were available to answer any questions. All forms were completed through face-to-face interviews in a private room at the hospital. Only literate women were included, as they needed to self-report on the SQOL. The privacy of participants was strictly maintained throughout the process.

### 2.6. Data analysis

The data obtained from the research were analyzed using the SPSS 25.0 statistical software. Normal distribution was assessed with the Kolmogorov–Smirnov test (*P* > .05). The Chi-square (χ²) test and *t* tests were used to compare the introductory characteristics of women with T1D and T2D, with results presented as frequencies (n), percentages (%), means, and standard deviations. The Turkish versions of the BDI and the SQOL were used to assess depression and SQoL, respectively. The *t* tests were employed to compare the BDI and SQOL scores between women with T1D and T2D. Pearson correlation analysis was used to examine the relationship between the mean total scores of the BDI and SQOL. Single linear regression analysis determined how depression scores affected SQoL in women with T1D and T2D, while multiple linear regression analysis identified factors predicting SQoL in women with diabetes. The evaluation was based on the coefficient of determination (*R*²). Data were analyzed at a 95% confidence interval, with significance set at *P* < .05.

#### 2.6.1. Ethical considerations

The study received ethical approval from the Izmir Katip Çelebi University Non-Interventional Clinical Research Ethics Committee (IRB: 0059/23.02.2023) and the hospital (Date: 05.04.2023). Additionally, permission was obtained from the researchers who validated the Turkish versions of the scales utilized in the study. Women diagnosed with diabetes were briefed on the study’s objectives, confidentiality measures, anonymity safeguards, and their rights as participants. Both written and verbal consent were obtained from those who voluntarily agreed to participate and met the predefined inclusion criteria. The research adhered to the Principles outlined in the Declaration of Helsinki.

## 3. Results

### 3.1. Population characteristics

Out of the total 440 women diagnosed with diabetes who participated in the study, 206 women with T1D and 234 women with T2D provided informed consent and completed the designated questionnaires. After propensity score matching, there were no significant differences in introductory characteristics such as age, education status, partner’s education status, employment status, income level, family type, number of children, and BMI between the groups (*P* > .05). The mean age for women with T1D was 35.39 ± 9.33 years, and for those with T2D, it was 45.02 ± 6.02 years. However, the mean age and number of children differed significantly between the groups (*P* < .001). Participants’ characteristics are detailed in Table 1.

**Table 1:** Introductory characteristics of the women with type 1 and type 2 diabetes

Variables	Type 1 diabetes (n = 206)		Type 2 diabetes (n = 234)		Total (n = 440)		Statistical analysis	
							*x*^2^/*t*	*P*
Age (yr) x̄ ± SD	35.39 ± 9.33		45.02 ± 6.02		40.51 ± 9.11		−2.661	< .001
Living child	1.22 ± 0.10		2.39 ± 1.31		1.84 ± 1.31		−1.647	< .001
	n	%	N	%	n	%		
Education status							1.684	.194
Under high school	92	44.7	119	50.9	211	48.0		
High school and above	114	55.3	115	49.1	229	52.0		
Partner’s education status							0.186	.666
Under high school	97	47.1	115	49.1	212	48.2		
High school and above	109	52.9	119	50.9	228	51.8		
Employment status							0.669	.414
Employed	104	50.5	109	46.6	213	48.4		
Unemployed	102	49.5	125	53.4	227	51.6		
Income level							4.615	.100
Low	19	9.2	34	14.5	53	12.0		
Middle	181	87.9	188	80.3	369	83.9		
High	6	2.9	12	5.1	18	4.1		
Family type							0.639	.424
Nuclear	191	92.7	212	90.6	403	91.6		
Extended	15	7.3	22	9.4	37	8.4		
BMI							3.288	.070
BMI ≤ 26	85	41.3	77	32.9	162	36.8		
BMI ≥ 26.1	121	58.7	157	67.1	278	63.2		

BMI = body mass index, SD = standard deviation, *t* = independent sample *t* test, x̄ = mean, χ2 = chi-squared test.

### 3.2. The score and comparison of SQOL and BDI

The scores and comparison of SQOL and BDI of the women with T1D and T2D are presented in Table 2. When women with T1D and T2D were compared, women with T2D had lower SQOL scores and higher BDI scores. There were significant differences between both groups in terms of both SQOL and BDI scores (*P* < .001; Table 2).

**Table 2:** The comparison of sexual quality of life and depression scores in women with type 1 and type 2 diabetes.

Variables	Type 1 diabetes (n = 206)	Type 2 diabetes (n = 234)	Statistical analysis	
	x̄ ± SD	x̄ ± SD	*t*	*P*
Sexual quality of life	72.39 ± 5.47	58.64 ± 6.58	5.535	< .001
Depression	11.53 ± 8.73	18.79 ± 6.44	−6.998	< .001

SD = standard deviation, *t* = independent sample *t* test, x̄ = mean.

### 3.3. Correlation of SQOL and BDI

The correlation between SQOL and BDI score is shown in Table 3. The women with T1D and T2D SQOL scores were strongly negatively correlated with BDI scores (respectively; *r* = −0.753, *r* = −0.837, *P* < .001; Table 3).

**Table 3:** The relationships between sexual quality of life and depression scores in women with type 1 and type 2 diabetes.

	Sexual quality of life			
	Type 1 diabetes (n = 206)		Type 2 diabetes (n = 234)	
	*r*	*P*	*r*	*P*
Depression	−0.753	< .001	−0.837	< .001

*r* = Pearson correlation coefficient.

### 3.4. The effect of depression score on quality of sexual life in type 1 diabetes and type 2 diabetes

The results of linear regression analysis for predicting SQoL of depression scores are presented in Table 4. The results of linear regression analysis showed that depression scores were a significant negative influencing factor on SQoL in women with diabetes (*P* < .001). It was determined that 1 unit increase in depression scores decreased SQoL scores by 2.2 units in type 1 diabetics (*B* = −2.196, *P* < .001) and 1.8 units in type 2 diabetics (*B* = −1.788, *P* < .001; Table 4).

**Table 4:** Results of regression analysis for predicting sexual quality of life of depression scores.

			Sexual quality of life												
	Type 1 diabetes (n = 206)								Type 2 diabetes (n = 234)						
	*B*	Standard error		β	*t*	CI 95%		*P*	*B*	Standard error	β	*t*	CI 95%		*P*
Constant	97.725	1.942		–	50.312	93.895	101.554		92.257	1.729	–	53.365	88.851	95.663	
Depression scores	−2.196	0.134		−0.753	−16.343	−2.461	−1.931	< .001	−1.788	0.077	−0.837	−23.300	−1.939	−1.637	< .001
Model summary	*R* = 0.753, *R*^2^ = 0.567, DW = 2.201, *P* = 0								*R* = 0.837, *R*^2^ = 0.701, DW = 1.695, *P* = 0						

B = unstandardized coefficient, β = standardized coefficient, CI = confidence interval, DW = Durbin–Watson, *R*^2^ = coefficient of determination.

### 3.5. Predictors of sexual quality of life in women with diabetes

A multiple regression analysis was conducted to identify factors independently associated with SQoL (dependent variable) in women with diabetes (n = 440). The analysis revealed that age (*B* = −0.291, *P* = .003), BMI (*B* = −2.747, *P* = .085), type of diabetes (*B* = −3.074, *P* = .091), and depression scores (*B* = −1.898, *P* < .001) were significantly related to SQoL (*P* < .05, *R*²=0.670), explaining 67% of the variance in SQoL (Table 5).

**Table 5:** Results of regression analysis for factors predicting sexual quality of life in women with diabetes (n = 440).

Independent variables	*B*	Standard error	β	*t*	CI 95%		*P*
Constant	106.208	3.630	–	29.258	99.074	113.343	< .001
Age	−0.291	0.099	−0.099	−2.955	−0.485	−0.098	.003
BMI (BMI ≥ 26.1)	−2.747	1.590	−0.049	−1.728	−5.872	0.377	.085
Type of diabetes (Type 2)	−3.074	1.815	−0.057	−1.693	−6.642	0.494	.091
Depression scores	−1.898	0.069	−0.807	−27.701	−2.033	−1.763	< .001
Model summary	*R* = 0.818, *R*^2^ = 0.670, DW = 2.003, *P* < .001						

Backward selected. Excluded Variables: Education status, Partner’s education status, family type, income level, employment status, living child.

B = unstandardized coefficient, β = standardized coefficient, BMI = body mass index, CI = confidence interval, *R*^2^ = coefficient of determination.

## 4. Discussion

Our study was the first study to explore the relationship between depression scores and SQoL in women with T1D and T2D. Diabetes was among the factors that negatively affected the SQoL of women.^[19–22]^ When the SQoL of women with TD1 and TD2 was compared, it was determined that the SQoL of women with T2D (low score) was worse than that of women with T1D (respectively 72.39 ± 5.47, 58.64 ± 6.58; *P* < .05). Previous studies have shown that T2D was among the factors that negatively affected the SQoL.^[19,23,44]^ The difficulties experienced by women with T2D regarding insulin regulation^[27,45,46]^ and the higher BMI of women with T2D negatively affected the SQoL.^[14,47]^ In addition, in our study, the mean age of women with T2D (45.02) was higher than that of women with T1D (35.39). According to the results of the regression analysis performed in our study, age harmed SQoL (*B* = −0.291; *P* = .003). In this context, it was determined that there was an inverse correlation between age and SQoL. Considering the negative effect of age on the SQoL in previous studies^[45,48]^ similar to our study, this result was among the other reasons that negatively affected the SQoL of women with T2D. In addition, the results of regression analysis in our study showed that T2D was among the factors that negatively affected the SQoL (*B* = −3.074; *P* = .091). For these reasons, it can be said that the SQoL of women with T2D and older age was worse in our study. In our study, it was observed that both women diagnosed with type 1 and type 2 diabetes experienced suboptimal levels of SQoL. Comparison with healthy counterparts revealed a lower SQoL among women with diabetes.^[22,49]^ This compromised SQoL adversely impacted various domains including marital satisfaction,^[22,50]^ body perception,^[47,51]^ and overall quality of life,^[50]^ while also contributing to psychological distress.^[46,52,53]^ Moreover, diabetes and its associated complications were found to potentially impede sexual performance in women due to factors such as diminished self-esteem, deteriorating health status, and disrupted social dynamics.^[50,54,55]^ Notably, depression emerged as a significant determinant of sexual dysfunction experienced by women with diabetes.^[46,52,53]^

Our study showed that depression scores of women with T2D (18.79 ± 6.44) were higher than women with T1D (11.53 ± 8.73; *P* < .05). Women with T2D had a higher risk of depression. Diabetes is among the important chronic diseases that increase the risk of depression.^[30–33]^ In previous studies, similar to our study, depression scores of patients with T2D were significantly higher.^[27,29,31]^ In addition, in the study by Mumtaz et al^[7]^ comparing the risk of depression in patients with T1D and T2D, 20% of patients with T1D and 40.6% of patients with T2D showed a risk of depression. It was reported that obesity and sedentary life,^[26,31,33]^ unhealthy diet, and nutritional problems^[32]^ were associated with T2D and increased the scores of depression. However, women were diagnosed with T2D at a certain period of their lives, not genetically. This process may have negatively affected their adaptation to the disease and increased their depression scores.^[29–31]^ In addition, it was reported that the type of treatment (Insulin was superior to oral diabetics.) received by women with T2D increased the risk of depression because it affected insulin regulation.^[27]^ Moreover, it was noted that women diagnosed with type 1 diabetes underwent more frequent monitoring and follow-up appointments compared to those with type 2 diabetes, attributed to patient volume in endocrine outpatient clinics in Türkiye. Consequently, diabetes nurses were prompted to identify signs of depression in women with type 1 diabetes at an earlier stage, facilitating prompt consultation with psychiatry. Hence, we posit that the higher depression scores observed among women with type 2 diabetes in our study may be attributed to these factors.

In our study, the SQOL and depression scores of women with both T1D and T2D found a strong negative correlation (*r* = −0.753; *r* = −0.837, respectively). In addition, the results of the regression analysis showed that 1 unit increase in depression scores of women with T1D decreased SQoL by 2.2 units (*B* = −2.196), and 1 unit increase in depression scores of women with T2D decreased SQoL by 1.8 units (*B* = −1.788). According to the results of regression analyses performed with 440 women with diabetes, a 1-unit increase in depression scores decreased the SQoL by 1.9 units (*B* = −1.898; *P* < .001). The results of our study showed that there was a negative relationship between depression scores and the SQoL of women with diabetes. Depression was among the most important causes of sexual dysfunctions experienced by women with diabetes. Previous studies focused on sexual dysfunctions experienced by women with diabetes^[14–17]^ and reported a relationship between sexual dysfunctions and depression.^[46,52,53]^ In a study conducted with women with T2D, it was concluded that women experienced sexual dysfunctions (decreased sexual desire and dyspareunia, etc) and sexual dysfunction increased the risk of depression.^[44]^ It was also shown that sexual dysfunction in women with diabetes had negative effects on the SQoL.^[14,19–21,34,45,56]^ In this context, we hypothesize that diabetes (in both groups) negatively impacts the SQoL in women and is associated with higher depression scores. Additionally, a reduced SQoL appears to increase the risk of depression.

Elevated depression levels in women with diabetes adversely affect both diabetes self-management and SQoL. The deterioration of sexual life quality and the increase in depression levels impaired both marital satisfaction and quality of life.^[22,50]^ These 2 parameters, which negatively affected each other, also negatively affected blood glucose monitoring and HbA1c results measured in 3-month periods in women with diabetes.^[57]^ Impaired insulin regulation in women with diabetes had negative consequences for body image.^[17,47,51]^ All these were causing women with diabetes to experience snowballing psychological and sexual problems and impair the management of diabetes.

The results of the regression analysis showed that BMI was among the parameters of the most significant 4-factor model. Because increasing BMI and obesity negatively affect self-esteem and body image and decrease the SQoL.^[17,47,51]^ It can be said that the SQoL of women with diabetes decreases due to both the negative effects of diabetes on sexual functions and sexual life and the negative body perceptions they experience with the increase in BMI. In addition, there was a complex relationship between diabetes, BMI, and obesity. High BMI increases the risk of diabetes. Furthermore, obesity was often associated with insulin resistance and type 2 diabetes. In overweight or obese individuals, body cells generally utilize insulin less efficiently, which may lead to insulin resistance. As a result, blood sugar levels were elevated and T2D could develop. Diabetes and obesity worsen each other. Increased BMI and obesity increased insulin sensitivity, made weight control difficult, and worsened diabetes control. Worsening diabetes control and increasing body mass index increased the risk of depression in women and negatively affected the SQoL.^[17,47,58]^

## 5. Limitations and strengths

Our study has some limitations. First, the study sample consisted of women with diabetes who came to the endocrine outpatient clinic. The second limitation was that the sample of the research was limited to one hospital. Thirdly, because the subject of the research was the quality of sexual life, which is highly confidential in Türkiye, some women were embarrassed and shy and did not complete the survey questions. On the other hand, the form and scales used were filled in based on self-report. The strengths of our study were that it was the first study in Türkiye to determine the SQoL and depression level of women according to the type of diabetes and to shed light on the relationship between them, were examined and reported in detail, the number of samples was reached in 7 months and this was a comprehensive sampling, the use of the forms of the scales adapted to Turkish culture in the study, women’s self-reporting of the questionnaires and attention to their privacy was important in terms of the reliability of the results and the quality of the research. All forms were administered through face-to-face interviews and completed by the patients on a daily basis, Monday through Friday. In the endocrine outpatient clinic of the university hospital where the study was conducted, women with diabetes were given training titled “Diabetes and its management” at certain intervals. Therefore, the women in the study sample were sexually active women who had been diagnosed with diabetes for at least 6 months. This increased the reliability of the results of our study. In addition, the research group consists of 6 academicians who are experts in their fields.

## 6. Conclusion

Our study revealed that women with T2D had worse SQoL and higher depression scores than women with T1D. In addition, increased depression scores in all women with T1D and T2D decreased the SQoL. Age, BMI ≥ 26.1, T2D, and depression score were among the factors that negatively affected the SQoL in women with diabetes. It is very important for healthcare professionals especially diabetes nurses to determine the depression level of women with T1D and T2D who come to the endocrine outpatient clinic and to evaluate their sexual health and SQoL. It is recommended that future studies should be carried out with larger and different sample groups in quantitative and qualitative studies.

## Acknowledgments

We thank the women who participated in the study.

## Author contributions

**Conceptualization:** Ekin Dila Topaloğlu Ören, Elif Ünsal Avdal, Funda Sofulu, Gökşen Polat, Gönül Düzgün, Gülseren Pamuk.

**Data curation:** Ekin Dila Topaloğlu Ören, Elif Ünsal Avdal, Funda Sofulu.

**Formal analysis:** Ekin Dila Topaloğlu Ören.

**Funding acquisition:** Ekin Dila Topaloğlu Ören, Elif Ünsal Avdal, Funda Sofulu, Gökşen Polat, Gönül Düzgün, Gülseren Pamuk.

**Investigation:** Ekin Dila Topaloğlu Ören.

**Methodology:** Ekin Dila Topaloğlu Ören.

**Supervision:** Ekin Dila Topaloğlu Ören, Elif Ünsal Avdal, Funda Sofulu, Gökşen Polat, Gönül Düzgün, Gülseren Pamuk.

**Writing – original draft:** Ekin Dila Topaloğlu Ören.

**Writing – review & editing:** Ekin Dila Topaloğlu Ören, Elif Ünsal Avdal, Funda Sofulu, Gökşen Polat, Gönül Düzgün, Gülseren Pamuk.
